# Glycemic Markers and Cardiometabolic Multiple Long-Term Conditions: Comparative Roles of Glycated Hemoglobin and Fasting Plasma Glucose in the English Longitudinal Study of Ageing

**DOI:** 10.1016/j.mayocpiqo.2026.100756

**Published:** 2026-09-12

**Authors:** Samuel Seidu, Setor K. Kunutsor, Kamlesh Khunti

**Affiliations:** a Leicester Real World Evidence Unit, Diabetes Research Centre, University of Leicester, Leicester General Hospital, Leicester, United Kingdom; b Hockley Farm Medical Practice, Leicester, United Kingdom; c Section of Cardiology, Department of Internal Medicine, Max Rady College of Medicine, University of Manitoba, Canada

## Abstract

**Objective:**

To examine and contrast the associations of glycated hemoglobin (HbA1c) and fasting plasma glucose (FPG) with the risk of cardiometabolic multiple long-term conditions (cMLTC) and evaluate their incremental predictive utility.

**Patients and Methods:**

The study analyzed 2233 adults (mean age 63; 1009 (45.2%) men) free of hypertension, coronary disease, diabetes, and stroke at baseline (wave 4; 2008-2009). Incident cardiometabolic multimorbidity (≥2 of these conditions) was assessed at wave 10 (2021-2023). Logistic regression, restricted cubic splines, and discrimination metrics evaluated predictors and predictive performance.

**Results:**

Over 12-15 years, 129 participants developed cMLTC (57.8 per 1000; 95% CI, 48.5-68.3). Restricted cubic splines showed continuous, dose–response relationships between HbA1c and FPG with cMLTC risk. HbA1c was strongly associated with cMLTC (per 1% increase, OR 1.99; 95% CI, 1.51-2.63; highest vs lowest tertile, OR 3.15; 95% CI, 2.00-4.94), whereas FPG associations were more modest (per 1 mmol/L: OR 1.39; 95% CI, 1.18-1.64; tertile: OR 1.98; 95% CI, 1.23-3.19) and attenuated after mutual adjustment. Adding HbA1c to established risk factors improved discrimination (ΔC-index=0.0344, *P*<.001) and fit (*P*<.001) more than FPG (ΔC-index=0.0171, *P*=.017; between-marker *P*=.050).

**Conclusion:**

In this nationally representative cohort of older adults in England, higher HbA1c and FPG levels were each associated with an increased risk of cMLTC and enhanced risk prediction. However, HbA1c emerged as a substantially stronger and more informative marker than FPG.

Cardiometabolic multimorbidity or cardiometabolic multiple long-term conditions (cMLTC), commonly defined as the co-occurrence of 2 or more cardiometabolic conditions such as diabetes mellitus, coronary heart disease (CHD), and stroke, has emerged as a major global public health challenge.^1^ The prevalence of cMLTC is increasing rapidly, driven by population aging, rising obesity, physical inactivity, unhealthy dietary patterns, and persistent socioeconomic inequalities.^2^^,^^3^ Individuals with cMLTC experience substantially higher risks of disability, reduced quality of life, healthcare utilization, and premature mortality compared with those with single cardiometabolic conditions.^4^, ^5^, ^6^ Importantly, cMLTC confers a risk that is greater than the sum of its individual components,^7^ underscoring the need to identify modifiable upstream risk factors and reliable tools for early risk stratification and prevention.

Established risk factors for cMLTC largely overlap with those for individual cardiometabolic diseases and include adiposity, hypertension, dyslipidemia, smoking, physical inactivity, and disturbances in glucose metabolism.^8^ Among these, dysglycemia plays a central role, not only in the development of type 2 diabetes but also in the pathogenesis of atherosclerosis, endothelial dysfunction, inflammation, and microvascular damage^9^^,^^10^—key biological pathways underpinning cardiometabolic disease clustering. Measures of glycemia, particularly fasting bin (HbA1c), are central to the screening, diagnosis, and monitoring management of complications, international guidelines recommend assessing glycemic status using FPG and/or HbA1c, the latter reflecting average blood glucose exposure over the preceding 2 to 3 months.^11^^,^^12^ Good glycemic control is critical for preventing complications and major adverse cardiovascular events and microvascular complications, and HbA1c is widely regarded as the cornerstone biomarker of long-term glycemic control.^13^

A robust and well-established body of evidence demonstrates strong, graded associations between glycemia and vascular outcomes. Higher levels of HbA1c and FPG are each consistently and independently associated with increased risks of vascular complications including CHD and stroke, in both people with and without diagnosed diabetes.^14^, ^15^, ^16^, ^17^, ^18^ Furthermore, several studies have shown that incorporating information on glycemic measures, particularly HbA1c, into prognostic models containing conventional cardiovascular risk factors is associated with modest but meaningful improvements in the prediction of first-onset cardiovascular disease (CVD).^14^^,^^19^

Despite growing interest in cMLTC as a distinct and clinically relevant outcome, the evidence base linking glycemic measures to the risk of cMLTC remains limited. Most existing studies have focused on individual cardiometabolic outcomes rather than their co-occurrence.^14^, ^15^, ^16^, ^17^ The few studies that have examined glycaemia in relation to cMLTC have predominantly been conducted in Asian populations and have often relied on composite indices, such as the HbA1c/high-density lipoprotein cholesterol (HDL-C) ratio, rather than evaluating HbA1c or FPG as independent exposures.^20^^,^^21^ Moreover, no studies to date have systematically assessed the potential utility of these glycemic measures in predicting the risk of developing cMLTC. Leveraging data from the English Longitudinal Study of Ageing (ELSA), a nationally representative prospective cohort of middle-aged and older adults in England, provides a unique opportunity to address these important gaps in the literature. The aim of this study is therefore to assess the nature and magnitude of the association between HbA1c levels and the risk of cMLTC and to evaluate the utility of HbA1c in predicting cMLTC risk. In parallel, we compare these associations and predictive performance with those of FPG within the same population, thereby providing direct evidence on the relative value of these two commonly used measures of glycemia for identifying individuals at high risk of cMLTC.

## Patients and Methods

### Study Population

This investigation was conducted and reported in accordance with the Strengthening the Reporting of Observational Studies in Epidemiology recommendations (Supplemental Appendix 1, available online at http://www.mcpiqojournal.org).^22^ Data were drawn from the ELSA, a continuing population-based cohort designed to be nationally representative of adults aged 50 years and older residing in private households in England.^23^ The ELSA cohort was established using participants recruited from the 1998, 1999, and 2001 Health Surveys for England,^23^ which served as the sampling frame for the first ELSA wave conducted in 2002-2003, enrolling 11,391 individuals aged 50 years or above. Participants have subsequently been followed at ∼2-year intervals across successive data collection waves.^23^ For the present analyses, wave 4 (2008-2009) was designated as the baseline assessment, and participants were followed prospectively through wave 10 (2021-2023). To focus on incident cMLTC, individuals with a history of hypertension, CHD, diabetes, or stroke at baseline were excluded.^24^, ^25^, ^26^, ^27^ Eligibility was further restricted to participants with complete information on HbA1c, FPG, relevant covariates, and cardiometabolic outcomes. After applying these inclusion and exclusion criteria, the final analytical sample comprised 2233 men and women. All participants provided written informed consent before participation, and ethical approval for ELSA was obtained from the London multicenter research ethics committee.

### Exposures, Covariates, and Outcome

Information on sociodemographic, lifestyle, and health-related factors was collected using a combination of structured interviews administered by trained nurses and standardized self-completion questionnaires. These instruments, which have been extensively used and validated in previous ELSA investigations, captured data on age, sex, smoking behavior, alcohol intake, physical activity patterns, and self-reported medical history.^23^^,^^24^^,^^28^^,^^29^ Alcohol consumption was assessed by asking participants how frequently they had consumed alcoholic beverages over the preceding 12 months. Smoking behavior was ascertained through a 2-stage process in which participants first reported whether they had ever smoked, followed by a question on current smoking status among those with a history of smoking.^29^ Physical activity was evaluated using a validated questionnaire that recorded engagement in light, moderate, and vigorous activities. In line with established ELSA classification criteria, participants were categorized into 4 groups: inactive (no reported physical activity), low (light activity only), moderate (moderate activity at least once per week), or high (vigorous activity at least once per week).^23^^,^^28^^,^^30^ Standardized physical examinations were conducted by trained nursing staff at mobile examination centers. These assessments included measurements of anthropometry, physical function, and cardiovascular parameters, and venous blood sampling for biochemical analyses. Height and weight were measured using calibrated equipment, with participants barefoot and wearing light clothing, and body mass index (BMI) was subsequently calculated as weight in kg divided by height in m^2^. Handgrip strength (HGS) was assessed using a Smedley dynamometer (Stoelting Co). Participants completed 6 grip strength trials—3 with each hand—starting with the dominant hand, with hands alternated and 1-minute rest periods between attempts. The maximum value achieved across all trials was used for analysis.^31^ Blood pressure was measured on the right arm using an automated Omron HEM 907 device (OMRON Healthcare Europe BV). Three readings were obtained during the assessment, and the average of the second and third measurements was used to derive systolic blood pressure (SBP) values.^32^ Venous blood samples were analyzed for HbA1c, FPG, and lipid parameters, including total cholesterol and HDL-C. Information on chronic health conditions, including hypertension, CVD, CHD, diabetes, and stroke, was obtained through participant self-reports of previous physician diagnoses collected during face-to-face interviews using structured questionnaires.^29^ cMLTC was defined at wave 10 as the presence of 2 or more of the following conditions: hypertension, CVD, diabetes, or stroke.^24^, ^25^, ^26^ Within ELSA, CVD was operationalized based on self-reported diagnoses of other heart disease, whereas myocardial infarction and stroke were treated as distinct outcomes. Although outcome ascertainment relied on self-report and detailed clinical adjudication of disease subtypes was not available, previous validation studies within ELSA and the closely related Health and Retirement Study have reported moderate agreement between self-reported diagnoses and clinically verified records for several cardiometabolic conditions.^32^^,^^33^

### Statistical Analysis

Baseline characteristics of participants were summarized using descriptive statistics. Continuous variables are presented as means with SDs or medians with interquartile ranges, as appropriate, based on distributional assessments using graphical methods and formal tests of normality. Categorical variables are reported as frequencies and percentages. Group differences were evaluated using independent-samples *t* tests for continuous variables and χ^2^ tests for categorical variables.

To examine the functional form of the associations between glycemic measures (HbA1c and FPG) and the subsequent risk of cMLTC, multivariable logistic regression models incorporating restricted cubic splines (RCSs) were fitted. In accordance with established recommendations for moderately sized samples, spline knots were placed at the 5th, 35th, 65th, and 95th percentiles of the exposure distributions.^34^ Evidence of nonlinearity was evaluated by comparing models containing only a linear exposure term with those including both linear and spline terms using likelihood ratio tests.^35^^,^^36^ Odds ratios (ORs) and 95% CIs for cMLTC were estimated using multivariable logistic regression. Covariate adjustment was performed sequentially across 4 models. Model 1 adjusted for age and sex. Model 2 further included smoking status, alcohol consumption, SBP, BMI, total cholesterol, HDL-C, and HGS. Model 3 additionally adjusted for physical activity. Model 4 included mutual adjustment for HbA1c and FPG to allow direct comparison of their independent associations with cMLTC risk. Given the strong correlation between HbA1c and FPG (Pearson r=0.62), potential multicollinearity was formally assessed prior to simultaneous modeling. Variance inflation factors were examined for all covariates, including HbA1c and FPG, and no evidence of problematic multicollinearity was observed (all variance inflation factors<2), indicating that collinearity was unlikely to materially influence the regression estimates. Covariates were selected *a priori* based on their established associations with cardiometabolic diseases, prior analyses within the ELSA,^24^, ^25^, ^26^^,^^28^^,^^31^ and their potential role as confounders.^37^ As spline analyses indicated broadly graded relationships, HbA1c and FPG were subsequently analyzed both as continuous variables (per unit increment) and as categorical variables based on tertiles. Continuous analyses were performed using the natural clinical units of each glycemic measure (HbA1c expressed as % and FPG as mmol/L). In addition, HbA1c and FPG were categorized according to established clinical thresholds to facilitate clinical interpretation.^38^^,^^39^ For risk prediction analyses, model discrimination was quantified using Harrell’s C-index for a base model containing conventional cardiometabolic risk factors (age, sex, smoking status, alcohol intake, SBP, BMI, total cholesterol, HDL-C, HGS, and physical activity), with and without the addition of HbA1c or FPG. Differences in C-indices and *P*-values were calculated using DeLong test for correlated C-indices.^40^ To complement discrimination metrics and evaluate improvements in overall model fit,nested models were also compared using the—2 log likelihood statistic, which is considered a sensitive approach for assessing incremental prognostic value.^41^^,^^42^ All statistical analyses were conducted using Stata MP version 18.0 (StataCorp), and a 2-sided *P* value <.05 was considered statistically significant.

## Results

### Baseline Characteristics

Baseline characteristics of the study population are summarized in Table 1, both overall and according to incident cMLTC status. Among the 2233 participants included in the analysis, the mean (SD) age at baseline was 62.5 (8.5) years, and women accounted for 54.8% of the cohort. The mean ± SD baseline levels of HbA1c and FPG were 5.7±0.5% and 4.82±0.74 mmol/L, respectively. Participants who went on to develop cMLTC during follow-up exhibited less favorable cardiometabolic profiles at baseline, including higher HbA1c and fasting glucose levels, higher BMI and SBP, higher triglyceride concentrations, lower HDL-C levels, and a greater prevalence of current smoking.

**Table 1:** Baseline Characteristics Overall and by cMLTC Status

Characteristic	Overall (N=2233)	No cMLTC (n=2104)	Yes cMLTC (n=129)	*P*
	Mean (SD), Median (IQR), or n (%)	Mean (SD), Median (IQR), or n (%)	Mean (SD), Median (IQR), or n (%)	
HbA1c, %	5.7 (0.5)	5.7 (0.4)	6.1 (0.9)	<.001
FPG, mmol/L	4.82 (0.74)	4.79 (0.66)	5.30 (1.45)	<.001
Age (y)	62.5 (7.1)	62.5 (7.2)	62.3 (6.4)	.74
Sex				.11
Male	1009 (45.2%)	942 (44.8%)	67 (51.9%)	
Female	1224 (54.8%)	1162 (55.2%)	62 (48.1%)	
Ethnicity				.94
White	2199 (98.5%)	2072 (98.5%)	127 (98.4%)	
Non-White	33 (1.5%)	31 (1.5%)	2 (1.6%)	
Current smoker				.014
No	1912 (85.6%)	1811 (86.1%)	101 (78.3%)	
Yes	321 (14.4%)	293 (13.9%)	28 (21.7%)	
Alcohol categories				.24
None	695 (31.1%)	650 (30.9%)	45 (34.9%)	
1-2 times/wk	584 (26.2%)	559 (26.6%)	25 (19.4%)	
3-4 times/wk	444 (19.9%)	420 (20.0%)	24 (18.6%)	
5 or more times/wk	510 (22.8%)	475 (22.6%)	35 (27.1%)	
Body mass index, kg/m^2^	27.6 (4.9)	27.4 (4.8)	30.1 (5.5)	<.001
Handgrip strength, kg	31.4 (11.1)	31.4 (11.0)	32.6 (12.1)	.23
Systolic blood pressure, mm Hg	130 (16)	130 (16)	138 (17)	<.001
Total cholesterol, mmol/L	5.89 (1.11)	5.90 (1.11)	5.66 (1.18)	.018
HDL cholesterol, mmol/L	1.61 (0.41)	1.62 (0.41)	1.45 (0.36)	<.001
Triglyceride, mmol/L	1.30 (1.00-1.80)	1.30 (1.00-1.80)	1.60 (1.20-2.40)	<.001
PA level				.69
Sedentary	56 (2.5%)	51 (2.4%)	5 (3.9%)	
Low	359 (16.1%)	337 (16.0%)	22 (17.1%)	
Moderate	1240 (55.5%)	1168 (55.5%)	72 (55.8%)	
High	578 (25.9%)	548 (26.0%)	30 (23.3%)	

cMLTC, cardiometabolic Multiple Long-Term Conditions; FPG, fasting plasma glucose; HbA1c, glycated hemoglobin; HDL, high-density lipoprotein; IQR, interquartile range; PA, physical activity.

### Associations of HbA1c and FPG With cMLTC

**Figure 1:**
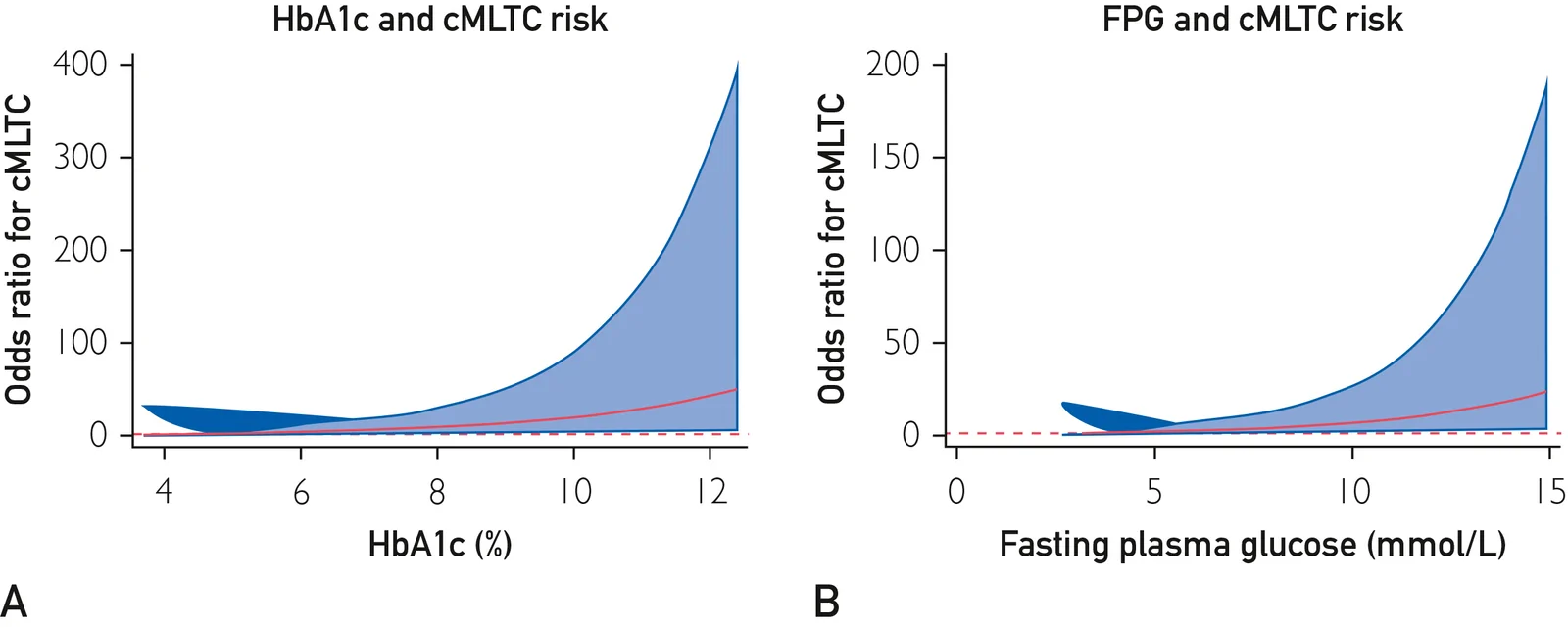
Restricted spline curves of the associations of HbA1c and FPG with the risk of cardiometabolic multiple long-term conditions. (A) HbA1c and (B) FPG. Shaded region represents the 95% confidence intervals for the spline model (solid red line).The model was adjusted for age, sex, smoking status, alcohol consumption, SBP, BMI, total cholesterol, high-density lipoprotein cholesterol, handgrip strength, and physical activity. cMLTC, cardiometabolic multiple long-term conditions; FPG, fasting plasma glucose; HbA1c, glycated hemoglobin.

During 12-15 years of follow-up, 129 participants developed CMM, corresponding to 57.8 per 1000 individuals (95% CI, 48.5-68.3). Among the 129 participants who developed cMLTC, incident hypertension (89.2%) and diabetes (49.6%) were the most frequently occurring component conditions, whereas myocardial infarction, stroke, and other cardiovascular disease were less common (Supplemental Table 2, available online at http://www.mcpiqojournal.org). A multivariable RCS curve showed the risk of cMLTC gradually increased continuously with increasing levels of HbA1c (Figure 1A).

**Figure 2:**
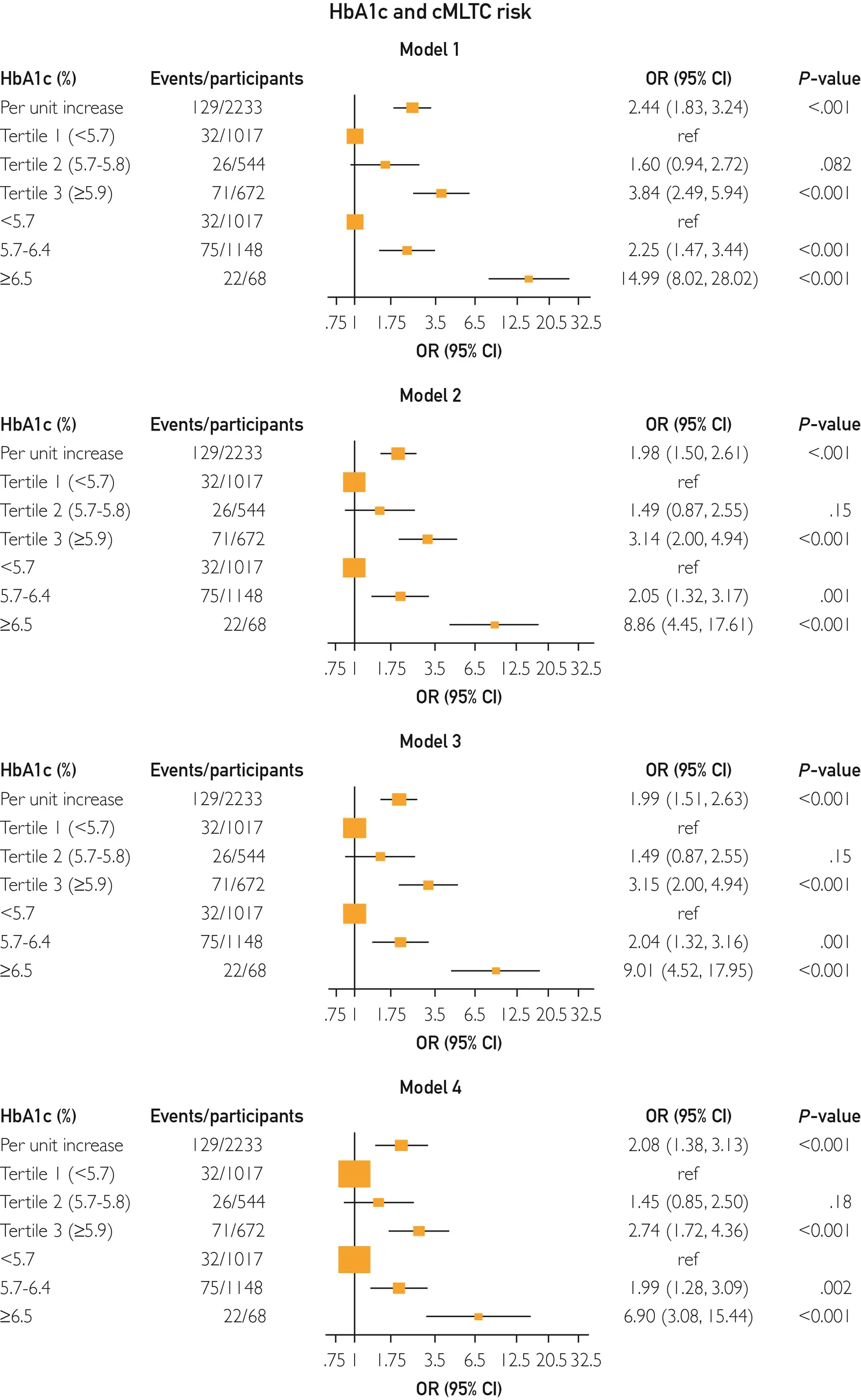
Associations of HbA1c with cardiometabolic multiple long-term conditions. Model 1: Adjusted for age and sex. Model 2: Model 1 plus smoking status, alcohol consumption, SBP, BMI, total cholesterol, high-density lipoprotein cholesterol, and handgrip strength. Model 3: Model 2 plus physical activity. Model 4: Model 3 plus FPG. cMLTC, cardiometabolic Multiple Long-Term Conditions; FPG, fasting plasma glucose; HbA1c, glycated hemoglobin; OR, odds ratio.

In multivariable logistic regression models adjusted for sex, smoking status, alcohol consumption, SBP, BMI, total cholesterol, HDL-C, and HGS (Figure 2-Model 2), higher HbA1c levels were strongly associated with an increased risk of cMLTC. Each 1% increment in HbA1c was associated with nearly a 2-fold higher odds of cMLTC (OR, 1.98; 95% CI, 1.50-2.61). This association was essentially unchanged after further adjustment for physical activity (OR 1.99; 95% CI, 1.51-2.63; Figure 2-Model 3) and remained robust following additional adjustment for FPG (OR, 2.08; 95% CI, 1.38-3.13; Figure 2-Model 4). Consistent findings were observed when HbA1c was examined categorically. Participants in the highest tertile of HbA1c had substantially higher odds of developing cMLTC compared with those in the lowest tertile, with adjusted ORs of 3.14 (95% CI, 2.00-4.94), 3.15 (95% CI, 2.00-4.94), and 2.74 (95% CI, 1.72-4.36) across Models 2 through 4, respectively. Similar patterns of association were evident when HbA1c was analyzed using established clinical thresholds (Figure 2).

**Figure 3:**
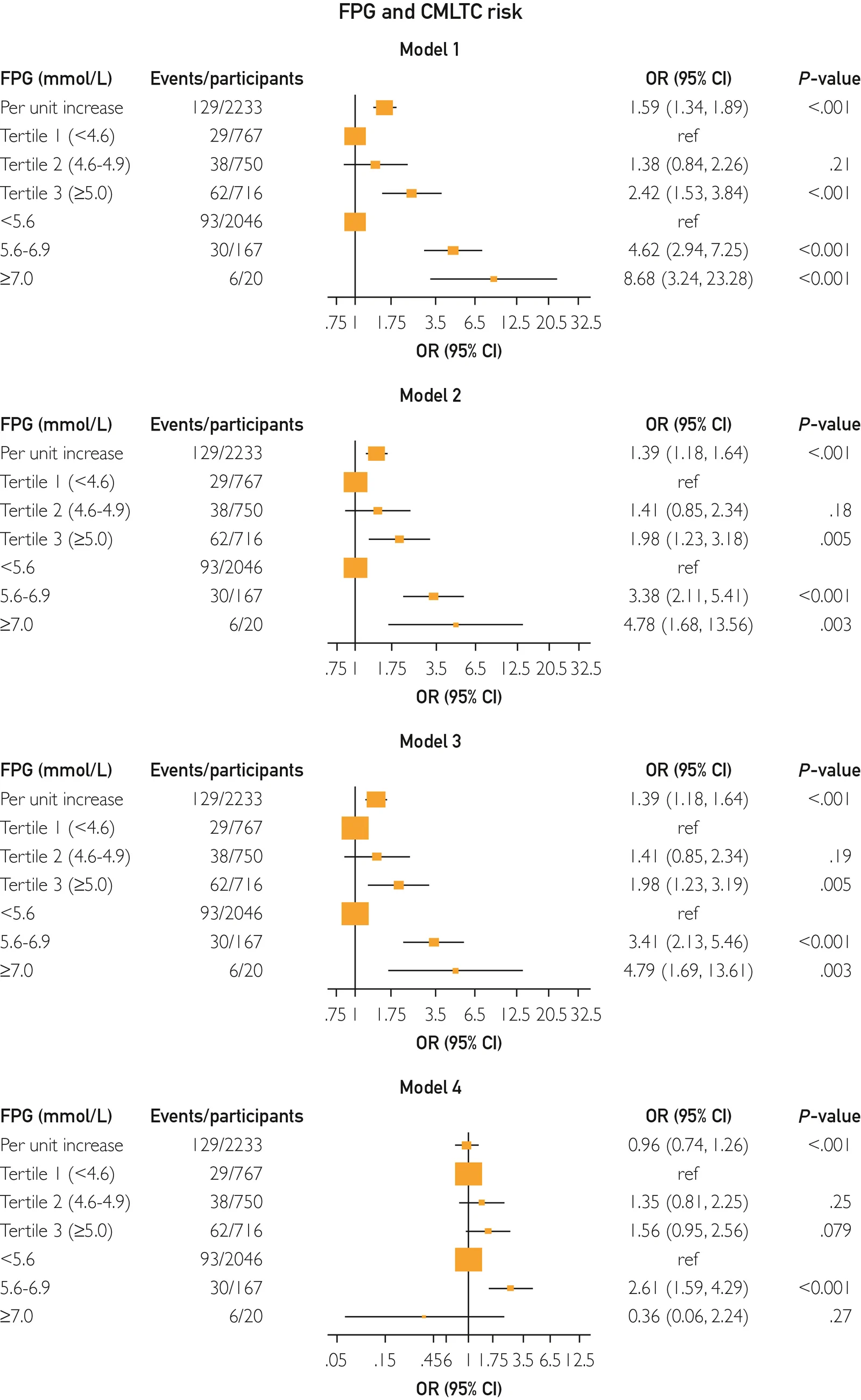
Associations of FPG with cardiometabolic multiple long-term conditions. Model 1: Adjusted for age and sex. Model 2: Model 1 plus smoking status, alcohol consumption, SBP, BMI, total cholesterol, high-density lipoprotein cholesterol, and handgrip strength. Model 3: Model 2 plus physical activity. Model 4: Model 3 plus HbA1c. cMLTC, cardiometabolic Multiple Long-Term Conditions; FPG, fasting plasma glucose; HbA1c, glycated hemoglobin; OR, odds ratio.

In parallel analyses of FPG, the risk of cMLTC increased continuously with increasing FPG levels (Figure 1B). Higher FPG levels were also associated with greater cMLTC risk in models adjusted for Model 2 covariates, with a 39% increase in odds per 1 mmol/L increment in FPG (OR, 1.39; 95% CI, 1.18-1.64; Figure 3). This association was unaffected by further adjustment for physical activity (OR 1.39; 95% CI, 1.18-1.64; Figure 3-Model 3). However, the association was markedly attenuated and no longer statistically significant after additional adjustment for HbA1c (OR, 0.96; 95% CI, 0.74-1.26; Figure 3-Model 4). When FPG was analyzed in tertiles, participants in the highest tertile had higher odds of cMLTC compared with those in the lowest tertile in Models 2 and 3 (OR, 1.98; 95% CI, 1.23–3.18 and OR, 1.98; 95% CI, 1.23-3.19, respectively), though this association was attenuated in Model 4 (OR, 1.56; 95% CI, 0.95-2.56). Analyses based on clinical FPG categories yielded qualitatively similar results (Figure 3).

Finally, when HbA1c and FPG were entered simultaneously into the same multivariable model, HbA1c reported a relatively stronger association with cMLTC risk than FPG. A formal Wald test comparing the corresponding regression coefficients confirmed a statistically significant difference between the 2 estimates (*P*=.019), indicating that HbA1c is the more informative glycemic measure in relation to cMLTC risk.

### Risk Prediction

The findings from the risk prediction analyses are presented in Table 2. A reference model incorporating established cardiometabolic risk factors (age, sex, smoking status, alcohol intake, SBP, BMI, total cholesterol, HDL-C, HGS, and physical activity) reported moderate discriminative ability, with a C-index of 0.7131 (95% CI, 0.6666-0.7596). The addition of HbA1c to this baseline model resulted in a meaningful enhancement in discrimination, increasing the C-index to 0.7475 (95% CI, 0.7023-0.7926). This represented an absolute improvement of 0.0344, which was statistically significant (*P*<.001). Consistent with this finding, inclusion of HbA1c was also associated with a significant improvement in overall model fit, as evidenced by the −2 log likelihood test (*P*<.001).

**Table 2:** Measures of Risk Discrimination on Addition of HbA1c and FPG to a cMLTC Risk Model Containing Established Risk Factors

Measure of discrimination	HbA1c	FPG
C-index (95% CI): established risk factors	0.7131 (0.6666-0.7596)	0.7131 (0.6666-0.7596)
C-index (95% CI): established risk factors plus exposure	0.7475 (0.7023-0.7926)	0.7302 (0.6843-0.7762)
C-index change (*P* value)	0.0344 (<.001)	0.0171 (.017)
*P* value for difference in -2 log likelihood	<.001	<.001

*P*-values for the change in C-index were calculated using DeLong test for correlated C-indices.

Abbreviations: cMLTC, cardiometabolic multiple long-term conditions; FPG, fasting plasma glucose; HbA1c, glycated hemoglobin; established risk factors include age, sex, smoking status, alcohol consumption, SBP, body mass index, total cholesterol, high-density lipoprotein cholesterol; handgrip strength, and physical activity level.

In comparison, adding FPG to the baseline model yielded a more modest, though still statistically significant, improvement in discrimination (ΔC-index=0.0171; *P*=.017), alongside a significant improvement in model fit based on the −2 log likelihood statistic (*P*<.001). When directly comparing the incremental gains in discrimination, the improvement attributable to HbA1c exceeded that observed for FPG by 0.0173, and this difference reached statistical significance (*P*=.050).

When HGS and physical activity, which are not typically captured in routine clinical practice, were excluded from the reference model, the gains in discrimination remained comparable (Supplemental Appendix 3, available online at http://www.mcpiqojournal.org).

## Discussion

In this prospective cohort of over 2000 middle-aged and older adults, higher levels of both HbA1c and FPG at baseline were associated with an increased risk of incident cMLTC over long-term follow-up. The risk of cMLTC increased progressively across the distributions of both glycemic measures; however, HbA1c reported a markedly stronger and more robust association than FPG, remaining relatively associated with cMLTC after multivariable and mutual adjustment. In risk prediction analyses, the addition of HbA1c to models containing established cardiometabolic risk factors produced greater improvements in discrimination and model fit than FPG, and these findings were consistent in subsidiary analyses using reference models that excluded HGS and physical activity, reflecting variables not routinely available in clinical records. Overall, HbA1c emerged as the more informative glycemic marker for identifying individuals at elevated risk of cMLTC.

A substantial body of evidence has reported that higher levels of HbA1c and FPG are associated with increased risks of individual cardiometabolic outcomes, including cardiovascular disease, diabetes complications, and mortality.^14^, ^15^, ^16^, ^17^ However, despite growing recognition of cMLTC as a clinically important outcome, we found no prior studies that have directly examined HbA1c or FPG in relation to the risk of cMLTC, nor any that have evaluated the comparative predictive utility of these glycemic measures for cMLTC. The limited existing evidence in this area comes from two studies based on the China Health and Retirement Longitudinal Study, which assessed the HbA1c/HDL-C ratio rather than HbA1c or fasting glucose as independent exposures.^20^^,^^21^ These studies reported a positive association between the HbA1c/HDL-C ratio and incident cMLTC, including evidence of a linear dose–response relationship, and showed that this association was independent of depressive symptoms.^20^^,^^21^ Although informative, these analyses relied on a composite index and were conducted in an Asian population, limiting direct comparability with our findings. In contrast, this study is, to our knowledge, the first to demonstrate that HbA1c and FPG are robustly associated with the development of cMLTC and to directly compare their relative strengths and predictive performance within the same population. By leveraging a large, nationally representative cohort of older adults in England and applying both etiological and risk prediction frameworks, our findings provide novel and clinically relevant evidence that HbA1c is a more informative glycemic marker than FPG for identifying individuals at increased risk of cMLTC.

Elevated levels of HbA1c and FPG reflect chronic dysglycemia, which promotes a range of pathophysiological processes implicated in the development and clustering of cardiometabolic diseases. These include insulin resistance, endothelial dysfunction, low-grade systemic inflammation, oxidative stress, and activation of pro-atherogenic and pro-thrombotic pathways, all of which contribute to vascular damage and metabolic deterioration over time.^9^^,^^10^ Persistent hyperglycemia also accelerates the formation of advanced glycation end products, further amplifying vascular injury and organ dysfunction.^43^ HbA1c may represent a stronger risk indicator and predictor of cMLTC than FPG because it captures longer-term glycemic exposure and day-to-day glucose variability, whereas fasting glucose reflects glycemia at a single time point and is more susceptible to short-term fluctuations and measurement variability.^44^ As a marker of cumulative glycemic burden, HbA1c may therefore more accurately reflect sustained metabolic stress and its downstream effects across multiple cardiometabolic systems, explaining its stronger and more consistent associations with cMLTC risk.

The findings of this study have important implications for both clinical practice and population health. First, the strong and continuous associations between HbA1c and the risk of cMLTC, together with its superior predictive performance compared with FPG, suggest that HbA1c may be a more informative marker for identifying individuals at elevated risk of developing multiple cardiometabolic conditions. These findings support the potential incorporation of HbA1c into routine clinical risk prediction systems to enhance early identification of individuals at high risk of cMLTC. Importantly, the consistency of the predictive gains observed even in models excluding HGS and physical activity—variables not routinely captured in clinical records - underscores the feasibility of integrating HbA1c into existing health care data infrastructures. From a public health perspective, these results highlight the potential value of HbA1c as a population-level screening and risk stratification tool for cMLTC. Given its relative stability, lack of requirement for fasting, and ability to capture long-term glycemic exposure, HbA1c is well suited for large-scale surveillance and prevention programs.^45^ Although the incremental improvement in discrimination afforded by HbA1c compared with FPG was modest, it was consistently observed across complementary measures of model performance. Whether this degree of improvement translates into meaningful clinical benefit warrants evaluation. Future studies should evaluate the performance and clinical utility of HbA1c-based risk prediction models using routine electronic health record data, such as the UK Clinical Practice Research Datalink, to assess their generalizability, implementation potential, and impact on clinical decision-making. Interventions aimed at improving long-term glycemic control as well as other cardiometabolic risk factors through lifestyle modification and, where appropriate, pharmacological treatment may not only reduce the risk of individual cardiometabolic diseases but also help curb the growing burden of multimorbidity in aging populations.

This study has several notable strengths. Foremost, the findings are novel. The analytical approach was robust, employing RCS models to characterize dose–response relationships, extensive adjustment for established and emerging cardiometabolic risk factors, and formal evaluation of predictive performance using validated measures of discrimination and model fit. Several limitations should also be considered. Owing to the observational design, causal inferences cannot be drawn. Information on exposures, outcomes, and some covariates, including chronic conditions and lifestyle behaviors, was largely based on self-report, which may be subject to misclassification, recall bias, or social desirability bias. Because incident hypertension was defined using self-reported physician diagnosis rather than serial blood pressure measurements, some participants with elevated blood pressure at baseline but without a prior diagnosis of hypertension may have been classified as free of hypertension at study entry and subsequently developed incident hypertension during follow-up. Information on antihypertensive medication use at baseline was not available, precluding confirmation that all participants classified as free of hypertension were untreated. Although individuals with a physician diagnosis of hypertension at baseline were excluded, some participants with undiagnosed or untreated hypertension may have remained in the cohort. Although a wide range of confounders was accounted for, the possibility of residual confounding from imprecisely measured or unmeasured factors cannot be excluded. The lack of detailed information on the timing of disease onset prevented the use of time-to-event analyses and limited assessment of disease sequencing. Our analyses were based on baseline measurements of HbA1c, FPG, and other covariates. Because these factors may change over time, the observed associations may not fully capture the effects of long-term trajectories or cumulative exposure. Future cohorts incorporating repeated measurements and time-varying analyses are needed to better characterize the dynamic relationships between glycemic status and the development of cardiometabolic multiple long-term conditions. In addition, the study population comprised older adults living in England, which may limit the generalisability of the findings to younger populations or other ethnic and geographical groups. Finally, the relatively small number of incidents cMLTC events may have reduced statistical precision for some estimates and limited the ability to conduct detailed subgroup analyses. Replication of these findings in larger and more diverse populations will be important to confirm their robustness and broader applicability.

## Conclusion

In this large, population-based study of older adults in England, higher levels of long-term and fasting glycemic markers were each associated with a progressively greater risk of developing cMLTC and improved risk stratification beyond conventional cardiometabolic factors. Notably, HbA1c reported a consistently stronger association and superior predictive performance compared with FPG. These findings underscore the clinical and public health importance of HbA1c as a practical and informative marker for identifying individuals at increased risk of accumulating multiple cardiometabolic conditions, with potential implications for earlier prevention and targeted intervention strategies.

## Potential Competing Interests

Dr Seidu is in receipt of speaker honoraria from AstraZeneca, Boehringer Ingelheim, Janssen, Lilly, MSD, Abbott, Novo Nordisk, SB Communications, OmniaMed Communications, Roche, Napp Pharmaceuticals, NB Medical and Amgen; advisory board honoraria from AstraZeneca, Lilly, Boehringer Ingelheim, Janssen, Abbott, MSD, Novo Nordisk, Takeda and Sanofi; educational grants from Boehringer Ingelheim, Lilly, Novo Nordisk and Takeda and conference registration and subsistence from Boehringer Ingelheim, Janssen, Lilly, Novo Nordisk, Abbott and Takeda. Dr Khunti has acted as a consultant and speaker for Novartis, Novo Nordisk, Sanofi-Aventis, Lilly and Merck Sharp and Dohme. He has received grants in support of investigator and investigator-initiated trials from Novartis, Novo Nordisk, Sanofi-Aventis, Lilly, Pfizer, Boehringer Ingelheim and Merck Sharp and Dohme. Dr Khunti has received funds for research, honoraria for speaking at meetings and has served on advisory boards for Lilly, Sanofi-Aventis, Merck Sharp and Dohme and Novo Nordisk. Setor K. Kunutsor has no conflict of interest.

## Ethics Statement

English Longitudinal Study of Ageing Wave 4 received ethical approval from the National Hospital for Neurology and Neurosurgery & Institute of Neurology Joint Research Ethics Committee on 12 October 2007 (07/H0716/48), and all participants provided written informed consent. Ethical approvals for the other waves in the ELSA project can be found on the website: https://www.elsa-project.ac.uk/ethical-approval.
