# Supplementary material for: Glycemic Markers and Cardiometabolic Multiple Long-Term Conditions: Comparative Roles of Glycated Hemoglobin and Fasting Plasma Glucose in the English Longitudinal Study of Ageing
Source: Mayo Clin Proc Innov Qual Outcomes. 2026 Sep 12;10(5):100756. doi: 10.1016/j.mayocpiqo.2026.100756 (PMC13587801; doi:10.1016/j.mayocpiqo.2026.100756)
Supplement: Supplementary file 1 [file mmc1.docx]

**Supplementary Material 1.** STROBE 2007 Statement—Checklist of items that should be included in reports of cohort studies

| **Section/Topic** | **Item #** | **Recommendation** | **Reported on page #** |
| --- | --- | --- | --- |
| **Title and abstract** | 1 | (*a*) Indicate the study’s design with a commonly used term in the title or the abstract | Page 1 |
|  |  | (*b*) Provide in the abstract an informative and balanced summary of what was done and what was found | Page 2 |
| **Introduction** | | |  |
| Background/rationale | 2 | Explain the scientific background and rationale for the investigation being reported | Page 4 |
| Objectives | 3 | State specific objectives, including any prespecified hypotheses | Page 4 |
| **Methods** | | |  |
| Study design | 4 | Present key elements of study design early in the paper | Study population |
| Setting | 5 | Describe the setting, locations, and relevant dates, including periods of recruitment, exposure, follow-up, and data collection | Study population |
| Participants | 6 | (*a*) Give the eligibility criteria, and the sources and methods of selection of participants. Describe methods of follow-up | Study population |
|  |  | (*b*) For matched studies, give matching criteria and number of exposed and unexposed | Not applicable |
| Variables | 7 | Clearly define all outcomes, exposures, predictors, potential confounders, and effect modifiers. Give diagnostic criteria, if applicable | Exposure, covariates and outcome |
| Data sources/ measurement | 8* | For each variable of interest, give sources of data and details of methods of assessment (measurement). Describe comparability of assessment methods if there is more than one group | Exposure, covariates and outcome |
| Bias | 9 | Describe any efforts to address potential sources of bias | Statistical analyses |
| Study size | 10 | Explain how the study size was arrived at | Statistical analyses |
| Quantitative variables | 11 | Explain how quantitative variables were handled in the analyses. If applicable, describe which groupings were chosen and why | Statistical analyses |
| Statistical methods | 12 | (*a*) Describe all statistical methods, including those used to control for confounding | Statistical analyses |
|  |  | (*b*) Describe any methods used to examine subgroups and interactions | Statistical analyses |
|  |  | (*c*) Explain how missing data were addressed | Not applicable |
|  |  | (*d*) If applicable, explain how loss to follow-up was addressed | Not applicable |
|  |  | (*e*) Describe any sensitivity analyses | Statistical analyses |
| **Results** | | |  |
| Participants | 13* | (a) Report numbers of individuals at each stage of study—eg numbers potentially eligible, examined for eligibility, confirmed eligible, included in the study, completing follow-up, and analysed | Study population |
|  |  | (b) Give reasons for non-participation at each stage | Study population |
|  |  | (c) Consider use of a flow diagram | Study design and population |
| Descriptive data | 14* | (a) Give characteristics of study participants (eg demographic, clinical, social) and information on exposures and potential confounders | Results; Table 1 |
|  |  | (b) Indicate number of participants with missing data for each variable of interest |  |
|  |  | (c) Summarise follow-up time (eg, average and total amount) | Results |
| Outcome data | 15* | Report numbers of outcome events or summary measures over time | Results |
| Main results | 16 | (*a*) Give unadjusted estimates and, if applicable, confounder-adjusted estimates and their precision (eg, 95% confidence interval). Make clear which confounders were adjusted for and why they were included | Results; Figures 1-3 |
|  |  | (*b*) Report category boundaries when continuous variables were categorized | Results; Table 1; Figure 1-2 |
|  |  | (*c*) If relevant, consider translating estimates of relative risk into absolute risk for a meaningful time period |  |
| Other analyses | 17 | Report other analyses done—eg analyses of subgroups and interactions, and sensitivity analyses | Results |
| **Discussion** |  |  |  |
| Key results | 18 | Summarise key results with reference to study objectives | Discussion |
| **Limitations** |  |  |  |
| Interpretation | 20 | Give a cautious overall interpretation of results considering objectives, limitations, multiplicity of analyses, results from similar studies, and other relevant evidence | Discussion |
| Generalisability | 21 | Discuss the generalisability (external validity) of the study results | Discussion |
| **Other information** |  |  |  |
| Funding | 22 | Give the source of funding and the role of the funders for the present study and, if applicable, for the original study on which the present article is based | Title page |

**Supplementary Material 2.** Frequency of incident component conditions among participants who developed cardiometabolic multiple long-term conditions

| **Component condition** | **Frequency** | **Percent** |
| --- | --- | --- |
| Hypertension | 115 | 89.2 |
| Diabetes mellitus | 64 | 49.6 |
| Other cardiovascular diseases | 33 | 25.6 |
| Stroke | 30 | 23.3 |
| Myocardial infarction | 29 | 22.5 |

**Supplementary Material 3.** Measures of risk discrimination upon addition of HbA1c and FPG to a cMLTC risk model containing established risk factors excluding handgrip strength and physical activity

| **Measure of discrimination** | **HbA1c** | **FPG** |
| --- | --- | --- |
| C-index (95% CI): established risk factors | 0.7139 (0.6673, 0.7605) | 0.7139 (0.6673, 0.7605) |
| C-index (95% CI): established risk factors plus exposure | 0.7480 (0.7027, 0.7932) | 0.7313 (0.6853, 0.7772) |
| C-index change (*p*-value) | 0.0341 (<.001) | 0.0174 (.013) |
| *p*-value for difference in -2 log likelihood | <.001 | <.001 |

CI, confidence interval; cMLTC, cardiometabolic Multiple Long Term Conditions; FPG, fasting plasma glucose; HbA1c, glycated hemoglobin; established risk factors include age, sex, smoking status, alcohol consumption, systolic blood pressure, body mass index, total cholesterol, and high-density lipoprotein cholesterol.
